# Future Strategic Priorities of the Swiss Decentralized Healthcare System: A COVID-19 Case Study

**DOI:** 10.3390/epidemiologia3020020

**Published:** 2022-05-16

**Authors:** Miriam Mi-Rim Lee Burger, Kaitlin Elizabeth Large, Yiqi Liu, Melissa Cigdem Coyle, Cherish Tariro Gamanya, Jean-François Etter

**Affiliations:** 1 Institute of Global Health, University of Geneva, 9 Chemin de Mines, 1202 Geneva, Switzerland; jean-francois.etter@unige.ch; 2 Global Studies Institute, Rue des Vieux-Grenadiers 10, 1205 Geneva, Switzerland; kaitlin.large@etu.unige.ch (K.E.L.); yiqi.liu@etu.unige.ch (Y.L.); melissa.coyle@etu.unige.ch (M.C.C.); cherish.gamanya@etu.unige.ch (C.T.G.)

**Keywords:** decentralization, Switzerland, healthcare, government, COVID-19, private–public collaboration

## Abstract

The COVID-19 pandemic exposed a multitude of vulnerabilities in Switzerland’s decentralized healthcare system and highlighted the urgent need to strengthen Switzerland’s capacity to respond to health crises and disease outbreaks. In this article, we draw on three distinct areas of analysis of the current functioning of the Swiss healthcare system to examine its strengths and weaknesses, which can serve as a basis for future considerations and strategic priorities. First, we analyze the different levels of nine non-pharmaceutical interventions (NPIs), as defined by the ETH KOF Stringency Index and implemented in the Swiss cantons of Zurich, Vaud, and Ticino, compared with the rate of positive COVID-19 cases, hospitalizations, and deaths. We find that there was no strong correlation between the severity of the nine non-pharmaceutical interventions implemented and lower rates of positive COVID-19 cases, hospitalizations, and deaths. Second, we examine the challenges of Switzerland’s decentralized healthcare system through a literature review and with empirical data obtained from semi-structured interviews with health professionals in Switzerland. We conclude our analysis with the role of central authorities during the COVID-19 pandemic. The results demonstrate that during a national emergency in Switzerland, taking into account other factors that influence the success of a pandemic strategy, there is an opportunity for a more unified, centralized response to reduce the social and economic toll of the pandemic without necessarily risking greater health damage. We recommend that the Swiss federal government use a combination of decentralized and centralized public health and policy approaches and promote greater private–public collaboration with direct communication channels among policymakers, public health stakeholders, and the public to improve pandemic preparedness and response.

## 1. Introduction

Switzerland (population 8.5 million) has a highly decentralized national health care system in which each of the country’s 26 cantons is independently responsible for funding, monitoring, coordinating, and promoting health care, although it is highly regulated by federal laws [1]. Sub-national (i.e., cantonal and municipal) government spending on health care accounts for more than 85% of total public health spending, and the Swiss federal government (SFG) is responsible for less than 20% of health care decisions [2], illustrating the high degree of autonomy of cantonal authorities. Proponents of health care decentralization argue that it improves efficiency, quality of services, coverage, and local accountability [3]. However, decentralization can also lead to uncoordinated responses to emerging public health problems, as illustrated by the COVID-19 pandemic, which can have social, health, and economic consequences.

The context of the COVID-19 pandemic provides a unique vantage point to examine and summarize both the benefits and limitations of a decentralized health care system. In the calendar year following the first confirmed COVID-19 case in Switzerland, federal measures were eventually replaced by cantonal regulations, resulting in a variety of responses and varying degrees of severity of COVID-19 cases across populations. The response of individual cantons was influenced by many factors, ranging from culture and politics to urban–rural disparities. As a result, inconsistent implementation of interventions led to some cantons experiencing greater economic, social, and health impacts than others, ultimately harming the Swiss health care system and the country as a whole. Most importantly, the government waited too long to implement the measures, resulting in high infection and mortality rates, even when adequate measures were eventually taken. Today, as Switzerland faces the threat of the Omicron variant and another COVID-19 wave, the search for valuable recommendations is as timely as ever.

Our paper aims to identify and recommend strategic priorities for the Swiss healthcare system to best prepare for and respond to current and future pandemics. Using the first year of the COVID-19 pandemic as a case study, we examine the inconsistent responses in non-pharmaceutical interventions (NPIs) implemented in the cantons of Zurich, Vaud, and Ticino, which represent the largest cantons in the German-, French-, and Italian-speaking regions of Switzerland, reflecting the diversity of the country. Next, we examine the importance of the private sector in the Swiss health care system, particularly the role of private insurers, test providers, and physicians, and the resulting strategies used during the pandemic. Finally, we explain the position and ability of the SFG to regulate the national health care system and weigh both the advantages and disadvantages of the extent of the SFG’s powers. By developing each of these arguments, we hope that lessons from the first year of the COVID-19 pandemic can provide an evidence-based impetus for developing recommendations for the strategic priorities of the Swiss health system in the future. We also hope to identify why the federal and cantonal governments could not or would not act more quickly and decisively.

## 2. Materials and Methods

We reviewed academic literature, grey literature from international organizations, and media reports published in recent years, especially in 2020 and 2021, to provide an overview of the health care system and the pandemic situation in Switzerland. The included sources were published in English and German.

To analyze the differences in NPI implementation between the cantons of Zurich, Vaud, and Ticino, we used publicly available data provided by the Swiss Economic Institute of the Swiss Federal Institute of Technology in Zurich (ETH) through its KOF Stringency Index, which captures the stringency of COVID-19 policies in Switzerland both at the national level and for all 26 cantons as of January 2020 [4]. The ETH KOF Stringency Index consists of the following nine sub-indices for lockdown policies: school closures, business closures, cancellation of public events, assembly restrictions, reduced public transport operations, regulations to stay at home, nationwide curfews, international travel restrictions, and public information campaigns. The nine sub-indices represented by the stringency index provide the correlation between the severity of pandemics in the cantons and the value of the index data. The dataset was truncated to include only the three cantons listed above, and the time period of interest was set from 3 July 2020 to 15 January 2021, reflecting the period during which cantons had the autonomy to implement measures as strictly as they saw fit. We selected the cantons of Zurich, Vaud, and Ticino for their representative size, population density, demographics, and sociocultural influence within each of the distinct language regions of Switzerland: Swiss-German, Swiss-French, and Swiss-Italian. As each canton represents a diverse region of Switzerland, together, they best highlight and reflect the overall differences in cantonal measures instituted during the COVID-19 pandemic within the country. The selected data points were then utilized to create our own data visualizations. To analyze the variance in COVID-19-positive cases, hospitalizations, and fatalities, we used publicly available data from the COVID-19 Info Switzerland dashboard (compiled from data provided by the Swiss Federal Office of Public Health and the respective cantonal health authorities). This dataset was also truncated to obtain data points only for Zurich, Vaud, and Ticino during the same time period (3 July 2020 to 15 January 2021) and utilized to create our own data visualizations [5].

To gain a more comprehensive understanding of the decision-making process, we also included the perspectives of various stakeholders and actors from the private sector, the public sector, academia, and the healthcare sector in different cantons of Switzerland. As of 15 December 2021, a total of 12 people were invited for in-depth interviews, of whom 6 participated (P1–P6), 5 did not respond, and 1 declined. Participants had a professional background in biomedical sciences, public health, epidemiology, and clinical medicine. Participants originated from Zurich, Vaud, and Ticino but also from institutions based in other cantons and with professional functions beyond cantonal borders. We followed the consolidated criteria for reporting qualitative research (COREQ) [6]. The Federal Act on Research involving Human Beings (Human Research Act) is not applicable to this study; thus, no official approval was required from the CCER committee of the Canton of Geneva. Data protection was respected in accordance with the principles of the Declaration of Helsinki. Before the interview, all participants (*n* = 6) were contacted by phone or email to schedule the interview and establish a relationship. Interviews were conducted by an author of this study who is an experienced and certified interviewer (MD). Participants did not learn additional details about the researchers’ goals and interests. Because of the COVID-19 pandemic measures, all interviews were conducted by telephone (*n* = 5) and video call (*n* = 1). Interviews were conducted in November and December 2021 in English and German, and they lasted between 20 and 60 min, with an average of 40 min. Notes were taken during and immediately after the interview to develop ideas for the study. To avoid any trace-back to the participants, the interviews were not recorded, and no transcripts were made.

## 3. Results

### 3.1. Disjointed Response: Zurich, Vaud, Ticino

#### 3.1.1. Overview of the Pandemic Situation in Switzerland

Switzerland is a demographically, linguistically, and politically diverse country. The small Alpine nation has four national languages: German, French, Italian, and Romansh, and 25% of the population are foreigners [7]. Each of Switzerland’s 26 cantons has its own constitution and is sovereign in almost all areas, including health care. Only in situations considered exceptional circumstances (such as public health crises, according to Article 6 of the 2012 Epidemics Act) can the SFG take control of the country’s preparedness for disease outbreaks and establish emergency rules [8]. Otherwise, the provision of emergency health care mechanisms is the responsibility of the 26 cantons [9]. As a result, in the year following the pandemic outbreak of COVID-19, pandemic-related regulations were implemented with varying degrees of severity at the cantonal level in Switzerland.

The first confirmed COVID-19 case in Switzerland occurred on 25 February 2020 in the canton of Ticino [10]. As of 8 December 2021, a total of 1.07 million COVID-19 cases have been confirmed nationwide, resulting in 11,625 fatalities [11]. The exact financial impact of the pandemic on the country’s economy cannot yet be calculated, but the Swiss Federal Statistical Office reports that Switzerland’s gross domestic product (GDP) fell by 2.4% in 2020 and the unemployment rate rose to 4% [12].

The SFG used its constitutional power to initially shift to a more centrist response, issuing a national decree on 28 February 2020, declaring the country in a “special situation”, and banning gatherings of more than 1000 people [13]. Switzerland maintained federal measures until 19 June 2020 (when the state of emergency ended under the Swiss Epidemics Act), and shortly thereafter, primary responsibility for implementing NPIs shifted to the cantonal level.

#### 3.1.2. Comparison of Zurich, Vaud, and Ticino

Using data and the scoring system from the Swiss Economic Institute ETH Zurich and the COVID-19 Info Switzerland dashboard, we compared the level of NPIs implemented in Zurich, Vaud, and Ticino between 3 July 2020 and 15 January 2021 and the number of COVID-19 confirmed cases, hospitalizations, and fatalities per 100,000 population in each canton, illustrating the COVID-19 severity of NPIs and the public health consequences in all three cantons. We then examined the relationship between the severity of the implemented NPIs and the severity of COVID-19 outcomes in the three cantons to determine whether correlations exist and whether each cantonal government acted appropriately in the interest of public health without causing undue social and economic harm.

ETH Zürich’s KOF Swiss Economic Institute’s Stringency Index uses the following sub-indicators as indices, with values between 0 (no measures) and 100 (full lockdown) to illustrate the level of lockdown policies over time and between cantons:

- Closure of schools, closure of workplaces, cancelation of public events, restriction of meetings, closure of public transportation, house arrest, restriction of domestic movement, international travel controls, public information campaigns, and face coverings. The nine sub-indices represented by the stringency index provide the correlation between the severity of pandemics in the cantons and the value of the index data.

**Figure 1:**
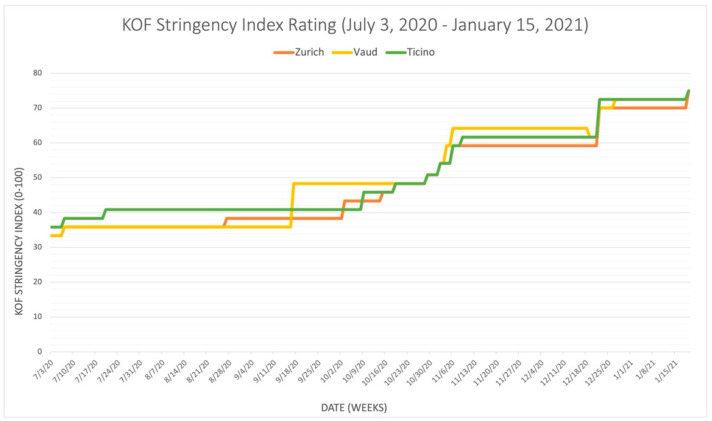
KOF Stringency Index rating of Zurich, Vaud, and Ticino.

Figure 1 uses daily KOF Stringency Index data to show the stringency of the measures implemented in the cantons of Zurich, Vaud, and Ticino during the period of autonomous cantonal jurisdiction (from 3 July 2020 to 15 January 2021), according to index scores.

Figure 1 illustrates that Ticino had the highest stringency index, closely followed by Vaud, while Zurich had the lowest stringency index during the period. Moreover, Figure 1 illustrates how all three cantons gradually increased the measures over time, at relatively similar times and by relatively similar values (given the probable imprecision of the index).

**Figure 2:**
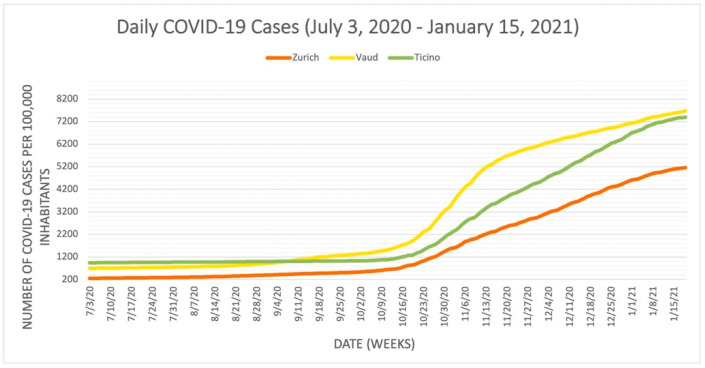
Reported daily COVID-19 cases in the cantons of Zurich, Vaud, and Ticino.

**Figure 3:**
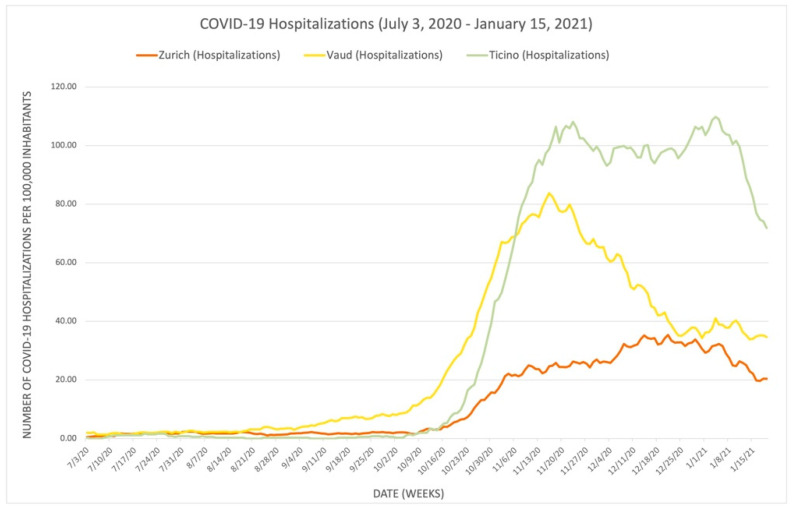
Reported daily COVID-19 hospitalizations in the cantons of Zurich, Vaud, and Ticino.

**Figure 4:**
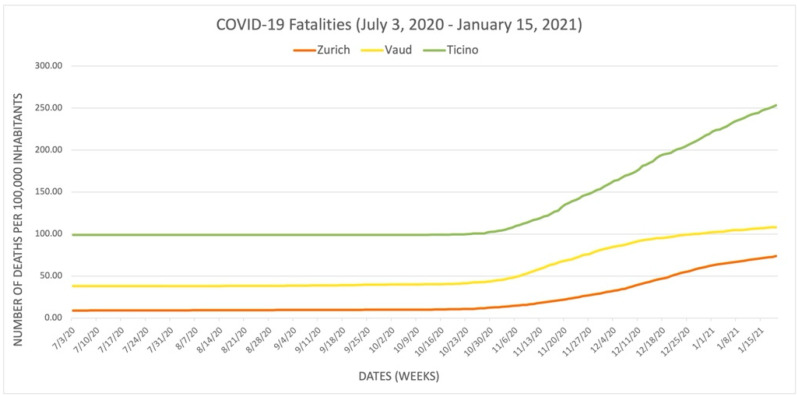
Reported daily COVID-19 fatalities in the cantons of Zurich, Vaud, and Ticino.

We juxtaposed Figure 1 with Figure 2, Figure 3 and Figure 4 to illustrate the correlation between daily stringency index values and daily COVID-19 cases, hospitalizations, and fatalities in Zurich, Vaud, and Ticino during the period (these results have been visualized in three separate graphs because of the different unit scales between the number of cases, hospitalizations, and fatalities). Figure 2, Figure 3 and Figure 4 use the publicly available data from the dashboard of COVID-19 Info Switzerland.

Figure 2, Figure 3 and Figure 4 illustrate that Vaud had the highest number of COVID-19 cases per capita, while Ticino had the highest number of severe COVID-19 positive outcomes per capita, including both hospitalizations and fatalities. All three figures also show that Zurich had the lowest COVID-19 outcomes in terms of daily COVID-19 cases, hospitalizations, and fatalities per capita during the period, although it also had the lowest stringency index rating. These figures illustrate that the stringency of the NPIs introduced (and, thus, the higher stringency index rating) does not necessarily correlate with lower rates of COVID-19 positive cases, hospitalizations, and fatalities when comparing cantons. Another interpretation of the contrast between Figure 1 and Figure 2, Figure 3 and Figure 4 suggests that the severity of the COVID-19 pandemic in the three cantons could be due to additional other factors because the three cantons adopted similar NPIs around the same time. These factors could also be variables for the lack of correlation between the stringency index numbers and pandemic outcomes in each canton, as they may influence the number of hospitalizations, ICU admissions, and fatalities. Several other factors include but are not limited to the pre-existing incidence and prevalence rates of COVID-19 in each canton; the specific demographic composition of the population; the wealth and the level of adherence of the population; the proximity of the most affected neighboring countries to each canton (such as Ticino’s proximity to Italy); the use of COVID-19 therapies such as steroids, oxygen, remdesivir, and other therapies; hospitalization criteria; the free movement of residents from one canton to another; and the actual impact of each NPI introduced, whose true effectiveness cannot be determined when combined with numerous other measures in an index. However, using the examples of Zurich, Vaud, and Ticino as a microcosm of relationships among cantons across the country, these factors demonstrate the potential for more coordinated action among cantons to mitigate the economic and social damage of the pandemic, as was the case in Zurich, without worsening COVID-19 health outcomes, as was the case in Vaud and Ticino. While decentralization can be observed at the national level, as demonstrated by the three selected cantons, it is also occurring in the private sector of the Swiss health care system. The following sections focus on the role of the private sector during the pandemic and illustrate the impact of decentralization on the COVID-19 response in Switzerland.

### 3.2. Private Sectors in the Healthcare System: Challenges or Advantages?

#### 3.2.1. The Importance of the Private Sector in the Swiss Healthcare System

The private sector plays an important role in most health care systems around the world. Challenges that have led the public sector to increasingly partner with the private sector typically include shifts in the burden of disease, particularly chronic noncommunicable diseases, demographic shifts, population shifts, political and economic instability, or crisis-related shortfalls in fiscal revenues. The private sector is often seen by governments as a solution to healthcare challenges, offering access to greater service capacity, responsiveness, management expertise, technology and innovation, and capital investment and financing [14].

In Switzerland, private insurance companies and health care provider associations share decision-making power with the government and the people, who can veto or demand reforms through public referendums in a direct democratic system. Legitimized civil society organizations of mandatory health insurance (MHI) providers represent and advocate for the interests of private insurers and private health care providers in the decision-making process [15]. This decentralized decision-making is supported by the corporatist tradition of the health care system, as responsibility for multiple regulatory functions has remained in the hands of the joint decision-making bodies of payers and providers. Overall, the private sector takes a prominent role, adding another layer of decentralization to the Swiss healthcare system.

The intensive involvement of the private sector also leads to potential challenges in the long-term transformation of the healthcare system, as it inevitably favors the interests of private companies to some degree. For example, due to the high-cost burden of inpatient care and the transition to outpatient care, Switzerland has experienced a twenty-year trend of reducing hospital capacity, which has had a negative impact on the country’s pandemic preparedness [16]. This is important during the COVID-19 pandemic because private sector involvement greatly influences decision-making processes and outcomes, leading to inconsistent responses across the cantons.

#### 3.2.2. Development of Private–Public Collaboration in Testing, Tracing, and Vaccination Policies

The COVID-19 pandemic placed both the SFG and cantonal decision-making powers on health policy and medical practice in an exceptional situation, as they were exposed to intense external political, media, and public pressure. From 28 February 2020 to 19 June 2020, the SFG had primary responsibility for setting policy, but the private sector played a critical role by providing materials and services. The private health sector does not operate on a pro bono basis, but most services provided by the private sector are reimbursed by the insurance system, which, in Switzerland, is also managed by private companies.

The Swiss National COVID-19 Science Task Force (NCS-TF) provided scientific support to the government in understanding and combating COVID-19 and identified innovation opportunities with respect to COVID-19 [17]. In this sense, the need for broader scientific participation in the pandemic process was met. However, the NCS-TF was integrated only after a long delay due to pressure from the scientific community in early April 2020, as reported by one interview participant (P6). The private sector maintained a relatively high degree of independence from the government, limiting the decision-making power of the SFG and possibly leading to delays in government response. The ever-changing regulations and policies on contact tracing, testing, and vaccination reflect this power dynamic.

##### Contact Tracing: The SwissCovid App

A notable collaboration between the private and public sectors is the emergence of “smart governance”. Guenduez et al. (2020) [18] reported that many historical measures used in previous pandemics are again being relied upon but have been improved through the use of intelligent technologies. For example, artificial intelligence is being used to better understand human behavior, enable rapid and effective contact tracing, demonstrate the effectiveness of interventions, track the evolution of the pandemic, and identify sites that are conducive to spread.

On 19 June 2020, the Swiss Parliament approved the launch of the SwissCovid app, which is based on a decentralized method for data processing. Switzerland’s technical universities, EPFL and ETH, have developed a digital tracking application that the Swiss population can use on a voluntary basis. The population’s data is encrypted, not stored centrally, and deleted periodically. The app does not use personal information such as names, mobile numbers, email addresses, or GPS data. Instead, it uses randomly generated codes that change every 15 min for identification. Data about encounters that could lead to infection is stored locally on individuals’ phones. Even if used in a centrally governed system, this ensures that no central authority can misuse this data. This application is also different from other European applications that have been withdrawn by EPFL and ETH due to data storage concerns. The project, led by Marcel Salathé and Carmela Troncoso at EPFL and ETH, demonstrates the possibilities of reconciling democratic principles with decentralized applications for tracking COVID-19 cases while respecting the constitutional right to privacy. While centralized states use smart technologies to control the population via top-down instructions, democratic systems rely more on decentralized solutions with citizen ownership [18]. The Swiss app COVID-19 is also an example of ideas developed by the public sector in Switzerland and then designed and produced by the private sector, illustrating the success of this fruitful collaboration. To date, parts of the protocol have been implemented in other countries’ COVID-19 tracking applications by private companies such as Google and Apple. The National Health Service application in the UK is one such example [19,20].

##### Testing

At the beginning of the pandemic (from 4 March to 22 April 2020), health insurers were only required to cover the cost of testing for coronavirus in patients with severe symptoms. In addition, the general cost-sharing rules applied, as the deductible payment and co-payment had to be borne by the insured person [10]. Despite the shortage of testing supplies at the time, this testing policy may also have contributed to the low rate of testing in the Swiss population, as it required patients to bear the costs associated with the test and, thus, may have hindered evaluation and control. To increase the supply of COVID-19 tests, the Swiss government worked with Swiss-based pharmaceutical companies, such as Roche, and asked them to convert some of their existing production capacity for other products to manufacture larger quantities of COVID-19 tests. This enabled a change in strategy on 23 April 2020, when testing was expanded to all individuals who had symptoms consistent with COVID-19, leading to a gradual increase in completed tests by August 2020 [16]. Then, in January 2021, SFG expanded its strategy by covering testing costs for people without symptoms and incentivizing more retesting [21]. As more test kits became available, testing strategies were further expanded until a large-scale vaccination campaign began [10]. The canton of Graubuenden was the first canton to systematically expand testing and demonstrate that preventive testing reduced the infection rate of COVID-19 by up to 50% [22,23].

In order to assess and improve the situation throughout Switzerland, it would have been critical to conduct a representative sampling of the population of each canton according to a standardized schedule throughout the course of the pandemic. Fehr (2020) [24] recommended the random sampling of 10,000 participants per 100,000 population, either by redrawing the sample each time or by testing the same participants each week. In order to obtain accurate data on the prevalence of the disease in the population, it is necessary to establish a rigorous allocation strategy that would put the test results in a meaningful context, with a realistic protocol for allocating these tests [25].

Less reliable tests (antigen tests, lateral flow tests) have also been offered as a widely available testing tool in most testing stations and for self-testing. Offering free PCR tests (laboratory-based tests) could be a more expensive but efficient solution to minimize bias, as they have higher sensitivity and specificity compared to antigen tests. In summary, governments would benefit from improving cantonal strategies for preventive testing, as previous strategies have not been orchestrated by the SFG. The cost of conducting national random testing would not have been comparable to the case where such testing programs had not been conducted and could have limited the growing fiscal damage and costs incurred [24,26].

The SFG’s decision to cancel free testing, beginning in November 2021, represents another change in strategy to get more individuals vaccinated. Tests offered by private healthcare providers now cost between CHF 15 and 195, depending on the type of test [27,28,29]. Since testing is now a financial burden for individuals and vaccination rates are increasing, there are no longer incentives to get tested regularly.

##### Vaccination

Another example of collaboration between the public and private sectors during the pandemic is Switzerland’s strategy for vaccine introduction. While the SFG is largely responsible for the procurement of vaccines, the implementation of the vaccination strategy from January 2021 has been based on the actual collaboration of the private health sector with the cantonal bodies. Vaccines are distributed centrally through the military pharmacy. The cantons have been responsible for the implementation of mass vaccination and have been free to decide which population group to vaccinate and in what order and how to do it. The cantons collaborated with public health bodies and private health care providers, such as general practitioners and family physicians, newly established vaccination centers, and hospitals, to conduct vaccination services at the community level. The FOPH and the Federal Vaccination Commission have recommended a vaccination strategy, and the SFC has not issued a mandatory priority list, although it would be legally possible to do so [10]. The fact that some cantons have been reluctant to implement a vaccination strategy has fueled the debate, as described in the previous sections.

In summary, the above examples show that the private and public sectors made progress during the pandemic in addressing challenges through collaboration and in a decentralized manner, as was the case with testing policy, a decentralized application for tracking, and the implementation of cantonal vaccination campaigns; test kits provided by private companies allowed the government to make decisions about scaling up testing policy, while the large-scale introduction of vaccines led to a reduction in free testing.

#### 3.2.3. Challenges in the Provision of Health Care Service during the Pandemic

Another important role played by the private sector was the provision of additional health care resources during the pandemic. According to federal law, the cantons are responsible for ensuring the availability of health care infrastructure, particularly hospitals, nursing homes, and emergency medical services. To this end, the cantons own the majority of hospitals [15]. Almost 70% of general inpatient acute care hospitals in Switzerland are publicly owned or subsidized [15]. Thus, when the pandemic began, cantonal governments reallocated primarily public resources to test and treat COVID-19 patients.

At the direction of the SFC, private and public hospitals also began to work closely together and retrain health personnel to deal with the outbreak. On one hand, the cantons were given the authority to require private health care providers to admit patients; on the other hand, they were instructed to install inter-cantonal cooperation with public and private hospitals that had intensive care units. Physicians were instructed not to perform non-urgent surgeries and treatments in light of the increasing number of COVID-19 hospitalizations. An example of the latter was the specialized role of the University hospitals, which were transformed into public COVID-19 treatment centers that received complex patient cases from neighboring cantons and hospitals, as also reported by one interview participant (P3) [30].

To maximize the available resources, most cantons invited private health care providers, especially general practitioners (GPs), to participate in the treatment of COVID-19 patients and to continue to care for their non-COVID-19 patients [31], as cantonal treatment capacity was at risk of being exhausted. Although these calls were not mandatory, many private health care providers chose to participate.

On the government side, however, there was a lack of coordination with the private health care providers involved. A study of private GPs in the canton of Vaud showed that the cantonal authority did not provide guidelines for dealing with suspected COVID-19 patients. They had to rely on themselves to equip themselves with personal protective equipment and decide how to deal with potential cases without the government recognizing their contribution [31].

Nevertheless, primary care physicians in primary care remained the primary source of medical advice for the population during the first wave of the pandemic COVID-19, and the most frequently consulted health care providers were GPs, specialists, pharmacies, the Internet, and accident and emergency departments—most of them from the private sector. Overall, people seemed to change their health care providers during the pandemic, with a tendency toward easily accessible and low-threshold medical services, as reported by one interview participant (P4) [32].

A study of 38 Swiss hospitals as part of the Quality Medicine Initiative showed the impact on patient care during the initial lockdown. Fewer patients were admitted to the hospital overall during the first six months of 2020 than during the same period in 2019, and the total number of ICU and ventilator cases was no higher than in 2019. Swiss hospitals experienced large revenue losses and additional costs, particularly due to the ban on elective surgery imposed by the SFC in March and April 2020 [33]. The infrastructural and staffing capacities of Swiss hospitals are not designed to provide additional services far beyond normal levels. The number of first-wave cases in very urgent situations (e.g., myocardial infarction, cerebral stroke) declined sharply, and it is likely that many patients have also foregone urgent hospital treatment. This is not desirable from either a health policy or a financial point of view [34].

The COVID-19 pandemic exacerbated the pre-existing challenges in the health sector. Long-term financial cuts were reflected in infrastructural challenges, deteriorating working conditions, an increasing number of unfilled positions, and an overload of existing staff in primary and secondary care. Although the reputation of the Swiss healthcare system is among the best in the world, the question is how to improve the overall pandemic preparedness of healthcare providers. The SFG and the cantons currently do not have a pandemic preparedness plan for COVID-19 in place.

### 3.3. Capacity of Central Authority

#### 3.3.1. Decentralization in the Swiss Healthcare System

The Swiss health care system is highly complex, combining aspects of controlled competition and corporatism in a decentralized regulatory framework shaped by the influences of direct democracy [15]. While canton-specific regulations allow for responsiveness to local priorities, the complexity raises questions about the need for federal coordination and the equity of the decentralized system. Tasks and responsibilities in the Swiss health care system are divided among the federal government, cantons, and municipalities. Each of the 26 cantons has its own constitution and is responsible for licensing health care providers, coordinating hospital services, promoting health through disease prevention, and subsidizing facilities and individual premiums [35]. Municipalities are primarily responsible for organizing and providing long-term care (nursing homes and home care services), health care in schools, and other social support services for vulnerable groups [35].

All measures to prevent and combat the spread of COVID-19 are based on the Federal Law on Epidemics, which was adopted by popular vote and, therefore, has a special democratic legitimacy [8]. However, there is evidence that the normal situation has not been adequately managed in recent times. Some cantons have preferred to prioritize and invest financial resources in more externally urgent health matters, such as cancer and dementia, while failing to meet national targets for stockpiling protective materials [36]. As a result, federal authorities did not use their surveillance powers to press for compliance with the national strategy, and, thus, the COVID-19 pandemic hit a country that was not as prepared as it should have been [37]. This section of the paper examines the advantages and disadvantages of centralized and unilateral/coordinated decisions in health care.

#### 3.3.2. Advantages of a Central Authority during COVID-19 and Future Pandemics

A strong central capacity has clear advantages when it comes to the ability to implement rapid and decisive action. In the United Kingdom, for example, measures to contain the spread and impact of coronavirus were enacted centrally on 3 March 2020, when the government unveiled its outbreak response plan [38]. At that time, there were 51 cases and no fatalities. In contrast, it took the SFC until 13 March to declare an “exceptional situation”, allowing it to take strict measures to prevent the spread of the virus. By that time, 1176 cases and 8 fatalities had been reported, and the medical community sharply criticized the government’s uncoordinated action [38]. While the declaration legitimized the SFC’s ability to lead the response, it had to continue to do so in close cooperation with its 26 cantons, which, until then, had led their own responses. Using this example of how Switzerland responded to the pandemic when it broke out and how the United Kingdom did, it is clear that a central authority is able to take effective action quickly. The opportunity cost of a disjointed response is much higher than a centralized system because of the time it takes to implement the measures. Another advantage of centralization is that decision-making in emergency situations can be timely. For example, when a pandemic breaks out, as in the case of COVID-19, measures to contain the disease can be implemented more easily because there are no delays in decision-making [39].

Most importantly, a centralized system of government ensures equitable development in a nation [39]. This is because development planning is done at the central level and implemented uniformly in all areas. For example, the health policy of a centralized government system might be to build a large hospital in each regional capital. Once that decision is made, it is implemented in a way that benefits the entire country. Finally, a centralized government provides discipline [39]. In a centralized system of government, the state has programs that must be implemented throughout the country, and every government employee is expected to implement the program. Even if the program needs to be changed, this must be approved by the central government before any change can be put into effect. Therefore, there is more discipline in a centralized government system than in a decentralized government system. In the context of the COVID-19 pandemic, this was a desirable approach, as measures such as travel restrictions contained the spread of the virus by controlling population movements in the 26 cantons.

#### 3.3.3. Disadvantages of a Central Authority during COVID-19 and Future Pandemics

A major disadvantage of having the SFG legislate is that decisions are likely to be made without a comprehensive local context of the situations in each canton; additionally, the government may not act quickly enough. For example, the requirement to wear masks was promulgated on 1 July 2020, two months after such regulations were adopted in France, Germany, Italy, and Austria [40]. Although the cantonal authorities and the Federal Office of Public Health were involved in the deliberations on the measures taken, these regulations could have been implemented easily at the cantonal level. Considering that different cantons had different COVID-19 infection and mortality rates, it could be said that waiting for consensus on the implementation of this measure was more of a failure because the government was slow to act. The wide gap between the cantons and the SFG became apparent during the pandemic. When the SFG announced its decision to introduce more national restrictions, such as limited numbers of people at social gatherings, the French-speaking cantons largely opposed this, while the German-speaking cantons supported it [41]. The basic principle of direct democracy, that all citizens participate in decision-making, contributes in some way to the institutional weakness of the Federal Council, which is a disadvantage.

## 4. Discussion

The pandemic has exacerbated some of the existing challenges in the Swiss health care system. Several additional factors influenced the development of the pandemic and may also have affected the outcomes in each canton differently. First, the pandemic highlights the lack of effective communication channels between the government and the population. For example, vaccination rates have remained relatively low in 2021. Possible explanations are that the population had to actively seek information on their own and lacked straightforward registration procedures, accessible locations, or convenient appointment windows. In the absence of a strong national immunization communication strategy, many factors have contributed to vaccination hesitancy: misleading news, conspiracy theories, distrust of authority, and downplaying the severity of COVID-19 have thrived, as do concerns about vaccine safety, side effects, and efficacy [42]. To counteract the negative influence of the media, information gaps and uncertainties, an improved government communication strategy is crucial.

Second, unsatisfactory working conditions for health care workers are also a challenge for the health care system. Deteriorating working conditions have led to up to 40% of nurses ending their careers prematurely, resulting in a shortage of qualified health care workers in intensive care units and clinics in general [43]. This may also have prevented an increase in ICU beds, leading to a reduction in pandemic preparedness. Some efforts have been made to address this issue for better pandemic preparedness, such as the November 2021 nursing initiative to have the nursing shortage addressed in legislation. The nursing initiative calls for Switzerland to strengthen the nursing profession and address the glaring shortage of skilled workers in several new constitutional articles [44]. However, more needs to be done to address the issue.

Finally, the problem of social inequality in access to health care is reflected in the low rates of testing and vaccination. People living in areas of low socioeconomic position were less likely to get tested but had a higher risk of testing positive, being hospitalized, or dying than people in areas of high socioeconomic position [45]. Governments and health care systems should address health inequities by taking action to reduce health inequities in response to the pandemic.

Switzerland’s response to the COVID-19 crisis was largely decentralized, allowing each canton to determine the level of NPIs to be implemented from 19 June 2020 to 15 January 2021. While decentralization allows cantons to adopt measures that are more appropriate for the local context, it can also create many complications and challenges for the country, especially during a global pandemic, where national coordination is essential. Analysis of the severity of NPIs implemented in Zurich, Vaud, and Ticino and the resulting COVID-19 cases shows that more stringent measures do not necessarily lead to better outcomes in COVID-19 cases (in terms of the number of cases, hospitalizations, and fatalities) but, rather, may contribute to worse economic and social outcomes, especially if these measures are implemented too late. This complexity raises questions about the need for coordination at the federal level and about the equity of the existing decentralized system.

To truly determine how severe the economic and social consequences have been in Vaud and Ticino compared to Zurich, a cost–benefit analysis would need to be conducted, taking into account the relevant direct, indirect, opportunistic, tangible, and intangible costs and benefits. Theoretically, the analysis should be conducted in a net benefit ratio, where the net costs are subtracted from the net benefits (B–C). If this results in a positive value, the current program of a decentralized emergency response is effective, and the investment is profitable. If the value is negative, the costs outweigh the benefits and Switzerland should adopt a different approach. In addition, it is necessary to calculate the margin or difference between the two numbers to establish a threshold for “readiness to change” or the number that adds sufficient value to change the system in times of emergency. This equation can also be applied to all cantons in Switzerland to collectively determine whether a centralized national response to the pandemic COVID-19 would have been more beneficial compared to the decentralized response that resulted, both in terms of economic and health impacts. The variables analyzed illustrate that health, social, and economic issues are intertwined and lead to various tradeoffs that must be weighed against each other.

The inconsistent response of the cantons parallels the inadequate communication and collaboration between public and private actors in health care, another result of Switzerland’s traditional regulatory and governmental systems. Differences in the application of national regulations at the local level may have contributed to the delay of coordinated testing and, more generally, pandemic policy. Statements, news reports, and live conferences by politicians and the Swiss COVID-19 Science Task Force provided observers with an unusual insight into the inner workings of the Swiss political system and the balance of power among national or regional cantonal authorities. When the national government issued orders to limit the spread of coronavirus in late February 2020, disagreements between the Swiss government and several of the 26 cantons became apparent. The cantons were slow to respond to SFG recommendations, slowing further collaboration with private healthcare providers.

It is crucial to understand the ways in which actors adhere to the federal system and how they cooperate with each other. In the case of the COVID-19 pandemic, strategic policymaking in a federal system proved more challenging. Ideally, policy could have been developed in harmony with the European Union, as Switzerland’s main trading partner, given that border crossers originate from France, Italy, and Germany. Above all, a coherent policy would promote the economic stability of Swiss companies, especially small and medium-sized enterprises, as well as multinational corporations [46].

The advantages of a central authority in managing the health care system during a pandemic seem to outweigh the disadvantages, but they can often coincide. For example, coordinated responses that allow for the timely implementation of policies and measures such as testing and vaccination can contain a pandemic. However, decisions made at the cantonal level have a local flavor and, therefore, better address the needs of the local community, such as closing restaurants and bars or shortening opening hours, which take into account the economic situation and population size of each canton and hopefully minimize the economic impact.

There is value in considering canton-specific needs, but in the case of a cross-border threat, a standardized, national response must be reinforced. Rather than being limited to one approach, a future strategic recommendation for a Swiss emergency response mechanism is to integrate elements of both approaches. In particular, if a cost–benefit analysis yields a negative result, there is an opportunity to revise the system. From our analysis, we conclude that the decentralization of the health care system is a major reason for the delayed pandemic response in Switzerland. One overarching question remains—how can the SFG and cantonal governments act more quickly and decisively in the event of a pandemic?

First, pandemic preparedness and response must be based on scientific evidence that may not be available in time. Second, more active communication is a critical element in preparing to manage public health emergencies. This includes not only communication with specific stakeholders but also communication with the scientific community and the general public and between science and government agencies at various levels to avoid latency. Reliable information from scientists to policymakers and timely communication to the public during a crisis are invaluable. Political scientist Adrian Vatter sees other influences on decision-making at the SFG level. First, the government’s involvement in the network of associations, interest groups, and lobbyists potentially leads to conflicts of interest. Second, each SFG member has multiple responsibilities, which can lead to uncoordinated procedures. Third, party interests come into play, as each member of the SFC is also a member of a political party [20].

Enabling direct communication channels between different governmental bodies, such as the SFG, cantons, and municipalities, will help ensure that centrally made recommendations are followed. At the same time, a mechanism must be established to hold the decision-making body accountable. As current communication between different levels of government is not fully transparent, it does not fully interact with health care providers or insurers that are part of the private sector. A new, less obstructive system would facilitate coordination between public and private actors, merging the two levels of communication. The lack of government structure in creating vaccine guidelines costs a lot of money and time due to the lack of infrastructure, and creating incentives could be a successful approach. In an emergency, the priority is to reduce the number of cases that require joint coordination between different factions to allow easy and quick implementation of the policy. While the accountability mechanism would need to incorporate the responsibility of each canton, it should not be implemented in a way that requires too much time for unanimity. Thus, the recommendation contains elements of both a decentralized and a centralized system, selecting those elements that provide the best value for coordinating an emergency response with low costs, high benefits, speed, clarity, and national coordination.

We also noted some limitations and opportunities for improvement in our study. Although not necessarily a weakness, the sample size limitation of the ETH KOF Stringency Index data means the results cannot be generalized to smaller, more rural cantons. Numerous other factors are thought to influence the results, and these factors could also explain the lack of correlation between the stringency index numbers and pandemic outcomes in each canton. Other factors that may influence the number of hospitalizations, ICU admissions, and fatalities include but are not limited to: the pre-existing incidence and prevalence rates of COVID-19 in each canton; the specific demographic composition of the population; the level of wealth and the level of adherence of the population; the proximity of the most affected neighboring countries to each canton (such as Ticino’s proximity to Italy); the use of COVID-19 therapies such as steroids, oxygen, remdesivir, and other therapies; the criteria for hospitalization; and the free movement of residents from one canton to another. In addition to these geographical, behavioral, biological, cultural, and sociodemographic aspects, climatic and physical factors, e.g., temperature, outdoor–indoor conditions, humidity, aerosol transmission, mucosal condition, and comorbidities, are relevant factors. All these factors pose limitations to our study.

However, it is impossible to extrapolate all of the above factors in one model, which was also confirmed in an interview (P1) in which the interviewee reported that various researchers believe that a direct correlation between the diverse catalog of COVID-19 measures and the outcomes are not currently possible. We, therefore, questioned whether it is possible to determine the true impact of individual NPIs, the actual effectiveness of which has not been combined with numerous other measures in an index, i.e., geographic, behavioral, climatic, biological, cultural, and sociodemographic factors, including the internal cultural divide in Switzerland. We, therefore, did not include these factors in our data analysis. Reported positive cases and warning indicators are currently the most reliable indicator for estimating subsequent hospitalization rates. Hospitalizations and ICU cases are even more reliable figures, but they are always about 7–14 days late and, therefore, limited in their predictive power. In addition, it may be relevant to assess how coherently the definition of the term “COVID-19 hospitalization” is used across the country and whether an international consensus on a definition has already been reached. Consequently, there may not yet be a rigorous definition of a “typical COVID-19 fatality case” because the clinical picture and spectrum of symptoms of COVID-19 are constantly evolving. Nevertheless, the combination of pandemic measures tested to date appears to be the best currently available to the Swiss government and cantons. The question remains of how to combine the dynamics of all the numbers (i.e., incidence, vaccine breakthrough rates, hospitalizations, ICU occupancy) into an extrapolation model for decision-making and how future variants of SARS-CoV-2 will affect this model.

## 5. Conclusions

Our analysis of the strategic actions taken by the Swiss government against the spread of COVID-19 illustrates how the variety of factors at play can influence the effectiveness of a strategy. Epidemiological, geographic, social, economic, cultural, medical, and political factors, the state of the healthcare system, and the availability of treatments and vaccines are all factors that influence the success of a strategy.

In addition, the bureaucratic tendency of the public sector to avoid risk and proceed in a deliberate manner, even when a national health crisis requires quick decisions and a degree of risk-taking, has slowed time-sensitive decision-making across the health care response. Quick decisions and a willingness to change the executive branch’s course when necessary are typical of an entrepreneurial mindset. This is not easy to find in the public sector, where routine processes predominate. Therefore, replacing bureaucratic processes with entrepreneurial efficiency could also significantly improve the management of future pandemics [47].

Finally, the COVID-19 pandemic exacerbated already existing challenges in the health care sector. Questions remain about how Switzerland can improve the overall pandemic preparedness of healthcare providers. A general shift to a national emergency strategy with a more centralized approach to protecting at-risk groups, aimed at suppressing viral spread but with less stringent measures, could potentially better address the long-term evolution of the situation.

## 6. Recommendations

As this paper focuses on future strategic recommendations for the Swiss health care system in light of future crises, it is also necessary to place the Swiss health care system itself in the context of global health. The following non-exhaustive list offers strategic suggestions for the health care sector:

1. Greater emphasis on the collaboration between the public and private sectors, particularly between health care providers and health insurers. Greater collaboration would benefit both population health and disease prevention immensely by leading to a more equitable sharing of the risks associated with poor health and the benefits associated with good health and healthy lifestyles. This would require a solutions-based approach that considers health care resources and the better distribution, accessibility, and execution of health care services. Intelligent technologies alone can provide significant opportunities in decentralized systems, but trust in the respective government and citizen participation is a prerequisite for the long-term success of future health care challenges. Synergies should not be compromised by government involvement in the network of political parties, associations, interest groups, and lobbyists.
2. Improved communication channels between governments, the public and private sectors, and the public. Different sectors operate under different constraints and regulations and approach problems in different ways, which can lead to impaired communication. Therefore, pandemic preparedness plans should establish direct communication channels between scientists and government agencies and between decision-makers and health care providers at different levels of government. This would enable active and comprehensive scientific communication that would improve timely decision-making and efficiency.
3. Optimization of the healthcare value chain. To improve health care at all system levels, it is imperative to reduce the costs associated with care. This may seem counterintuitive, but it calls for the right incentives rather than emphasizing the wrong incentives. However, if risks and benefits are shared between providers and health insurers, providers could improve care—for example, by including more health-promoting services inside and outside hospitals—and reduce the number of disease services that the current system overwhelmingly provides. Prioritizing preventive care models and long-term cost-saving initiatives would not only reduce morbidity and mortality and save money later but would also start building strong provider–recipient relationships earlier and strengthen personal health accountability. Examples of trust-building structures could include college curricula with a preventive care focus or the provision of low-threshold community frameworks and health services that support long-term health behavior change [48]. In this sense, understanding and optimizing the healthcare value chain could help reduce patient admissions, prevent ICU overcrowding, provide sufficient treatment capacity, and reduce overall costs for hospitals and emergency departments.
4. Promotion of the role of social determinants and health literacy. Prevention and basic health literacy should be more widely funded and promoted throughout society. Population compliance is greatly influenced by the level of health literacy that individuals bring to community discussions. Health literacy encompasses comprehension, numeracy, critical media literacy, and digital literacy and can contribute to a more comprehensive understanding of lifestyle or health choices and their impact on mental health, chronic disease management, substance use disorders, pregnancy, parenting, and younger population behaviors. Promoting health literacy could support overall confidence in and acceptance of public health interventions but may also increase demand for health care services.
5. Application of the United Nations Sustainable Development Goals as a project catalyst and future-directed vessel. The SDGs provide powerful conceptual guidance for greater collaboration across sectors and more rigorous global standards for a sustainable future in all areas of work and life, particularly health, business, and policy. Given the unmet need to develop a pandemic preparedness plan, the context of the SDGs could be an opportunity to combine sustainable, long-term national programs with powerful requirements for emergency situations.

## Data Availability

Publicly available datasets were analyzed in this study. The dataset for Figure 1 is available from ETH Zurich, KOF Swiss Economic Institute, KOF Stringency Indices [4], and can be found here: [https://kof.ethz.ch/prognosen-indikatoren/indikatoren/kof-stringency-index.html] (accessed on 17 November 2021). The dataset for Figure 2, Figure 3 and Figure 4 is available from the dashboard COVID-19 Info Switzerland [5] and can be found here: [https://www.covid19.admin.ch/en/overview] (accessed on 15 January 2022).

## Abbreviations

GDP	Gross domestic product
NPI	Non-pharmaceutical intervention
SFG	Swiss Federal Government
P1–P6	Interview participants No. (n = 1–6)
SFC	Swiss Federal Council
ETH	Swiss Federal Institute of Technology in Zürich
MHI	Mandatory health insurance
THE	Total health expenditure
NCS-TF	Swiss National COVID-19 Science Task Force
FOPH	Federal Office of Public Health
EPFL	École Polytechnique Fédérale de Lausanne
GP	General practitioner
SDGs	Sustainable Development Goals

## References

[1] Isabelle Sturny International Health Care System Profiles: Switzerland. https://www.commonwealthfund.org/international-health-policy-center/countries/switzerland.

[2] James C., Beazley I., Penn C., Philips L., Dougherty S. (2019). Decentralisation in the Health Sector and Responsibilities across Levels of Government: Impact on Spending Decisions and the Budget. OECD J. Budg..

[3] Harvard T.H., Chan School of Public Health Decentralization International Health Systems Program. https://www.hsph.harvard.edu/international-health-systems-program/decentralization/.

[4] KOF Stringency Indices. https://kof.ethz.ch/en/forecasts-and-indicators/indicators/kof-stringency-index.html.

[5] COVID-19 Switzerland|Coronavirus|Dashboard. https://www.covid19.admin.ch/en/overview.

[6] Consolidated Criteria for Reporting Qualitative Research (COREQ): A 32-Item Checklist for Interviews and Focus Groups|The EQUATOR Network. https://www.equator-network.org/reporting-guidelines/coreq/.

[7] European Comission Population: Demographic Situation, Languages and Religions: Switzerland. https://eacea.ec.europa.eu/national-policies/eurydice/content/population-demographic-situation-languages-and-religions-115_en.

[8] The Federal Assembly of the Swiss Confederation SR 818.101—Federal Act of 28 September 2012 on Controlling Communicable Human Diseases (Epidemics Act, EpidA). https://www.fedlex.admin.ch/eli/cc/2015/297/en.

[9] Wyss K., Lorenz N. (2000). Decentralization and Central and Regional Coordination of Health Services: The Case of Switzerland. Int. J. Health Plan. Manag..

[10] Federal Office of Public Health FOPH: Coronavirus: Vaccination. https://www.bag.admin.ch/bag/en/home/krankheiten/ausbrueche-epidemien-pandemien/aktuelle-ausbrueche-epidemien/novel-cov/impfen.html.

[11] Johns Hopkins University & Medicine Switzerland—COVID-19 Overview—Johns Hopkins. https://coronavirus.jhu.edu/region/switzerland.

[12] Office F.S. Marked Decline in GDP in 2020 Following COVID-19—Swiss National Accounts 2020|Press Release. https://www.bfs.admin.ch/bfs/en/home/statistics/catalogues-databases/press-releases.assetdetail.18344126.html.

[13] Burci G.L. Jennifer Hasselgard-Rowe Switzerland’s Response to the COVID-19 Pandemic|IHEID. https://www.graduateinstitute.ch/communications/news/switzerlands-response-covid-19-pandemic.

[14] Clarke D. Technical Series on Primary Health Care. The Private Sector, Universal Health Coverage and Primary Care. World Health Organization 2018. https://www.who.int/docs/default-source/primary-health-care-conference/private-sector.pdf?sfvrsn=36e53c69_2.

[15] De Pietro C., Camenzind P., Sturny I., Crivelli L., Edwards-Garavoglia S., Spranger A., Wittenbecher F., Quentin W., World Health Organization, Regional Office for Europe, European Observatory on Health Systems and Policies (2015). Switzerland: Health System Review 2015.

[16] Desson Z., Lambertz L., Peters J.W., Falkenbach M., Kauer L. (2020). Europe’s COVID-19 outliers: German, Austrian and Swiss policy responses during the early stages of the 2020 pandemic. Health Policy Technol..

[17] Federal Office of Public Health FOPH COVID-19 Task Force. https://www.bag.admin.ch/bag/en/home/das-bag/organisation/direktionsbereiche-abteilungen/krisenorganisation-covid-19.html.

[18] Guenduez A. Wie Smarte Städte Pandemien Bekämpfen Können. www.ncbi.nlm.nih.gov/pmc/articles/PMC7481040/pdf/35114_2020_Article_272.pdf.

[19] Swiss Tracing App Goes on Trial|ETH Zurich. https://ethz.ch/en/news-and-events/eth-news/news/2020/05/swiss-covid-app.html.

[20] Longchamp C. How to Make the Federal Council More Weatherproof. https://www.swissinfo.ch/eng/politics/how-to-make-the-federal-council-more-weatherproof/46208704.

[21] Canton of Zurich, General Secretariat: Erweiterte Teststrategie: Umsetzung im Kanton Zürich. https://www.zh.ch/de/news-uebersicht/medienmitteilungen/2021/02/erweiterte-teststrategie-umsetzung-im-kanton-zuerich.html.

[22] Kanton Graubünden: Graubünden Beweist: Wiederholtes Testen Senkt Corona-Ansteckungen Deutlich. https://www.tagblatt.ch/news-service/leben-wissen/auswertung-der-massentests-graubuenden-beweist-wiederholtes-testen-senkt-corona-ansteckungen-deutlich-ld.2134271.

[23] Kanton Glarus: Corona-Flächentests Erfolgreich Gestartet. https://www.gl.ch/public-newsroom/details.html/31/news/21499.

[24] Fehr E. Test the World to Make It Safer Place. https://testtheworld.org.

[25] Riachi I. https://towardsdatascience.com/8-key-points-you-might-want-to-think-about-before-sharing-that-next-covid-19-stat-with-your-friends-812c134de124.

[26] Fehr E. Neue Zürcher Zeitung: Economist Ernst Fehr Accuses Policymakers of Making Decisions Based on Insufficient Data—And He Makes a Suggestion (Video). https://www.nzz.ch/panorama/coronavirus-oekonom-fordert-repraesentatives-testing-ld.1548239?reduced=true.

[27] (2021). University of Zurich, Department of Global Health, Zentrum für Reisemedizin. https://coronazentrum.uzh.ch/de/testen.

[28] Ab Heute Sind Coronatests Nicht Mehr Gratis. https://www.srf.ch/news/schweiz/neues-testregime-ab-heute-sind-coronatests-nicht-mehr-gratis.

[29] Federal Office of Public Health Coronavirus: Health Insurance Arrangements. https://www.bag.admin.ch/bag/en/home/krankheiten/ausbrueche-epidemien-pandemien/aktuelle-ausbrueche-epidemien/novel-cov/regelung-krankenversicherung.html.

[30] Giachino M., Valera C.B.G., Velásquez S.R., Dohrendorf-Wyss M.A., Rozanova L., Flahault A. (2020). Understanding the Dynamics of the COVID-19 Pandemic: A Real-Time Analysis of Switzerland’s First Wave. Int. J. Environ. Res. Public Health.

[31] Cohidon C., El Hakmaoui F., Senn N. (2021). The role of general practitioners in managing the COVID-19 pandemic in a private healthcare system. Fam. Pr..

[32] Giezendanner S., Fischer R., Hernandez L.D., Zeller A. (2021). The use of health care during the SARS-CoV-2 pandemic: Repeated cross-sectional survey of the adult Swiss general population. BMC Public Health.

[33] Westerhoff C., Kuhlen R., Schmithausen D., Graf R., Winklmair C. Effekte der COVID-19 Pandemie auf Die Stationre Versorgung. https://doi.emh.ch/saez.2021.19616.

[34] PWC: Whitepaper 3.0 zur Berechnung des Finanziellen Schadens für Schweizer Spitäler und Kliniken Infolge von COVID-19. https://www.pwc.ch/de/publications/2020/20201216_COVID19_Whitepaper_3.0.pdf.

[35] Tikkanen R., Osborn R. International Profiles of Health Care Systems. https://www.researchgate.net/publication/347011106_International_Profiles_of_Health_Care_Systems_2020/link/5fd7897292851c13fe865e6a/download.

[36] Bruhin E., Wüthrich A. (2014). Spectra—Gesundheitsförderung und Prävention.

[37] Belser E.M., Mazidi S. Does Swiss Federalism Need Oxygen Treatment after Been Hit by the COVID-19 Crisis?. https://uacesterrpol.wordpress.com/2020/06/02/does-swiss-federalism-need-oxygen-treatment-after-been-hit-by-the-covid-19-crisis/.

[38] Gaskel J., Stoker G. Centralised or Multi-Level: Which Governance Systems Are Having a ‘Good’ Pandemic?. https://blogs.lse.ac.uk/politicsandpolicy/governance-systems-covid19/.

[39] Morton C. The Merits of a Centralized System of Government. https://www.virtualkollage.com/2016/12/the-merits-of-centralization-system-of-government.html.

[40] The Local: Why Did It Take Switzerland so Long to Make Masks Compulsory?. https://www.thelocal.ch/20200701/why-are-coronavirus-masks-still-not-required-in-switzerland/.

[41] Klaunzer P. Cantons Threaten Rebellion against Swiss Mini-Lockdown Plans. https://www.swissinfo.ch/eng/cantons-threaten-rebellion-against-swiss-mini-lockdown-plans/46213134.

[42] Hitchman S. Reasons for Not Getting Vaccinated against COVID-19 in German-Speaking Switzerland: An Online Survey among Vaccine Hesitant 16–60 Year Olds. https://psyarxiv.com/hnzke/.

[43] Tschannen P. Pflegeinitiative: Es Braucht Mehr Als Nur Mehr Ausbildung. Neue Zürcher Zeitung. https://www.nzz.ch/meinung/pflegeinitiative-es-braucht-mehr-als-nur-mehr-ausbildung-ld.1651964.

[44] (2021). Pflegeinitiative. https://www.pflegeinitiative.ch.

[45] Riou J., Panczak R., Althaus C.L., Junker C., Perisa D., Schneider K., Criscuolo N.G., Low N., Egger M. (2021). Socioeconomic position and the COVID-19 care cascade from testing to mortality in Switzerland: A population-based analysis. Lancet Public Health.

[46] Geiser U. How the Virus Puts the Swiss Political System to Test. https://www.swissinfo.ch/eng/federalism-and-covid-19_how-the-virus-puts-the-swiss-political-system-to-test/45646244.

[47] Simonian H. How the Private Sector Could Help to Fight the Next Pandemic. https://www.avenir-suisse.ch/en/how-the-private-sector-could-help-to-fight-the-next-pandemic/.

[48] Global Social Prescribing Alliance. https://whis.world/gspa.

